# A quantitative, multicentre, longitudinal study of patient experiences after gynaecological day surgery

**DOI:** 10.1002/nop2.1403

**Published:** 2022-10-09

**Authors:** Anne‐Marie Gran Bruun, Katrine Svensen, Elin Johansen, Thor‐David Halstensen, Anette Gustavsson, Ann‐Chatrin Linqvist Leonardsen

**Affiliations:** ^1^ Department of Nursing and health sciences University of Southeastern Norway Borre Norway; ^2^ Department of Anaesthesia Vestfold Hospital Trust Tønsberg Norway; ^3^ Department of Anaesthesia Ringerike Hospital Hønefoss Norway; ^4^ Department of Health, Welfare and Organization Østfold University College Østfold Norway; ^5^ Department of Anaesthesia Østfold Hospital Trust Østfold Norway

**Keywords:** anaesthesia, exercise intervention, patient perspectives, quality assurance

## Abstract

**Aim:**

The aim of this study was to explore patients' experiences after gynaecological day surgery one and 30 days postoperatively, as well as potential factors influencing these experiences.

**Design:**

The study had a multicentre, quantitative, longitudinal design.

**Methods:**

The study was conducted in three different hospitals' day surgical unit and included patients undergoing gynaecological surgery in general anaesthesia. We used a questionnaire including the European Quality of Life tool (EQ5D3L), the Quality‐of‐Recovery‐15 questionnaire (QoR‐15) and items relating to patient experiences, the first day (T1, *n* = 444) and 30 days (T2, *n* = 193) after surgery. Data were collected in the period March 2019 to March 2020.

**Results:**

Results show that patients mainly had positive experiences and ranged quality of recovery high, even though some areas needed improvement. Patient scores on the QoR‐15 relating to their experiences 24 h postoperative were rated higher at T1 than at T2. Twenty per cent of the respondents experienced complications such as infection, haemorrhage and pain. About 1/5 of these contacted healthcare services, and three per cent was hospitalized. EQ5D score was the only factor that made an statistically significant impact on patients' experiences with quality of recovery (*R* ^2^ .169, *F* = 82.87). However, this effect was weak.

## 1 INTRODUCTION

The increasing amount of elder people and people with long‐lasting diseases increase the need for medical treatment, and hereby the pressure on healthcare services (St Sauer et al., 2015). Hospitals are over‐booked, and there is a lack of healthcare professionals to take responsibility for the increasing patient population (Bureau of Labor Statistics, 2017). Internationally, there has been a political and medical emphasis on increasing the amount of patients undergoing day surgical procedures, keeping them out of hospitals. Twenty years ago, 25 per cent of all elective operations were day surgery; today, this accounts for about 60 per cent (Lieng et al., 2013; The Norwegian Directorate of Health, 2016). Day surgery, or ambulatory surgery, is defined as elective surgery in hospitalized patients, who are discharged to home the same day. The typical day surgery patient is admitted for 4–6 h, with slightly longer stays in more complicated procedures. Due to progresses in anaesthesiological and surgical techniques, more complex and complicated patients and procedures may be conducted as day surgery (Quemby & Stocker, 2014). The goal of day surgery is that patients reach the same physiological status as before the procedure as soon as possible. Planning for early discharge, day surgery implicate challenges related to treatment of negative consequences due to the surgery, such as pain and postoperative nausea and vomiting (PONV) (Fehrman et al., 2007), as well as prevention of complications. A recent study in Norway showed that 16 per cent of day surgery patients experience PONV (Stjernberg et al., 2018). This study did not include information about, for example, age, weight or comorbidity.

Patient experiences offer another, acknowledged dimension of quality indicators, such as the incidence of infections or re‐admissions. In addition, positive patient experiences have been positively associated with healthcare quality, patient safety and efficacy (Grøndahl et al., 2011). The terms “satisfaction,” “preferences” and “experiences” are used interchangeably, despite their different meaning. Satisfaction can be defined as the gap between patients' expectations and patients' experiences. Hence, patient‐reported experiences are judged less subjective than reported satisfaction (Grøndahl et al., 2011; Salisbury et al., 2010). Information about patient experiences is important, since this give an opportunity to meet patients' expectations, to administer and supervise healthcare performance and support strategic decision‐making in order to improve services (Al‐Abri & Al‐Balushi, 2014). Patient satisfaction after day surgery has been associated with clinical outcomes such as 30‐day re‐admission to hospital, as well as on postoperative complications related to the surgical procedure (Jaensson et al., 2017; Quemby & Stocker, 2014). A study from 2004 showed that, despite that 95% of day surgical patients were satisfied both at discharge and 30 days after surgery, *complete* satisfaction was present in only 75 per cent of patients at discharge and reduced to 62 per cent after 30 days, and thirty per cent of patients had moderate‐to‐severe pain after the procedure (McGrath et al., 2004). A study from 2011 showed that gender and experienced anxiety had a statistically significant association with reported Quality of Recovery (QoR‐ see methods section) (McIntosh, 2011).

Studies have revealed several areas that need improvement in day surgical patients. For example, one study indicated that incomplete information increases the experience of pain and anxiety and a feeling of lack of help after discharge (Mitchell, 2015). Other factors with a negative impact on patient satisfaction presented are “type of surgery” (Stessel et al., 2015), “young age” and “employment status = not working” (Brix et al., 2017), “duration of the procedure,” “female gender” and “laparoscopic cholecystectomy” (Stessel et al., 2015). “Pain” has been reported as the most common reason for contact with healthcare services after discharge in addition to a need for further information and instructions (Boissard et al., 2018; Brix et al., 2017). Despite medical progress and development due to, for example, medication and surgical or anaesthesiological techniques, day surgery still have great challenges with ensuring positive patient experiences. Most of the studies internationally have focused on patient satisfaction and related to a specific surgical procedure, and mainly directly after discharge (the next day).

Research on day surgery outcomes is limited, especially related to patients' experiences longitudinally and research that link patients' experiences to, for example, age or comorbidity. Such knowledge is essential when planning, developing and improving healthcare services. Hence, the purpose of this study was to explore patients' experiences after gynaecological day surgery the first postoperative day and 30 days after surgery, as well as potential factors influencing these experiences.

## 2 METHODS

The study had a quantitative, longitudinal, multicentre approach. The study adheres to the STROBE checklist presented in the Enhancing the QUAlity and Transparency Of health Research (EQUATOR) guidelines. Consent was granted from the Norwegian Committee for Medical and Health Services Research and the Norwegian Centre for Research Data. Participation was based on guidelines for ethical research in the Declaration of Helsinki (World Medical Association, 2015), and on willing, informed consent.

### 2.1 Setting and participants

The study was conducted in three different hospitals with day surgery services. Table 1 gives a description of the three locations.

**TABLE 1:** Study setting

	Østfold	Tønsberg	Ringerike
Catchment area	310,000	250,000	80,000
Number of procedures	5,000	5,000	1,645

*Note*: Catchment area = approximately number of inhabitants in the hospitals' catchment area. Number of procedures = number of day surgical procedures provided as of 2017.

To be able to describe patient experiences in relation to the same procedure, as well as to be able to reach the sample size at all three locations, we chose to focus on gynaecological procedures that were conducted as day surgery in the three hospitals, respectively. All patients fulfilling the inclusion criteria were invited to participate.

### 2.2 Inclusion criteria

- Able to give their written consent to participate.
- Age 18 years or older.
- Able to read and understand the Norwegian language.
- Planned gynaecological procedure in general anaesthesia.

### 2.3 Exclusion criteria

- Psychiatric disease or cognitive impairment (as documented psychiatric or dementia diagnosis).
- Re‐admission in 72 h.
- Elective abortions.

Together with a statistician, we calculated the needed sample size based on being able to detect statistically significant differences in QoR‐15 total scores between hospitals. Assuming a level of significance = .05, a power of 80 per cent, standard deviation (SD) = 0.3 and minimal difference in interest of 5 per cent on the QoR‐15 total score, we would need to include 100 patients from each hospital.

### 2.4 Data collection

Personnel in the three day surgery units and the postoperative anaesthesia care unit (PACU) were provided information about the study. All patients received written information about the study before the admittance, as well as oral information by the nurse anaesthetist/study nurse in the day surgery unit before the procedure. The study was conducted in two phases:

- T1 – 24 h after the procedure study nurses at the day surgery unit interviewed patients by telephone, according to the phase 1 questionnaire. Here, answers were plotted into a pre‐programmed iPad at each location, with direct transfer to a safe research service: TSD (Tjenester for sensitive data, services for sensible data) at the University of Oslo, Norway.
- T2 – 4 weeks after the procedure patients were instructed to answer the phase 2 questionnaire, following a link sent by email. Patients who did not have email/ a computer were given the opportunity to complete a paper‐based questionnaire, which was returned to the project leaders' office address in a pre‐stamped envelope. The questionnaire was made in the University of Oslos' “Nettskjema” (web‐form), with direct transfer to TSD.

### 2.5 Phase 1 questionnaire

The questionnaire in the first phase consisted of four parts:

1. Socio‐demographics including gender, age, living arrangement (living alone, not living alone), civil status (married/in a relationship, single, widow/widower, girl−/boyfriend), employment status (working/not working) and educational background (elementary school, upper elementary school, university/university college).
2. Self‐reported health‐related quality of life, using the validated EuroQoL 5 dimension 3 level version questionnaire, EQ5D3L. The questionnaire is divided into five different areas: mobility, self‐care, usual activities, pain/discomfort and anxiety/depression. Responses are scored according to three levels, where 0 = no problems, 1 = some problems and 2 = severe problems. The EQ5D3L score can be used as an overall index score by assigning weights to each dimension according to the Europe VAS value set (range 0–1). In addition, patients completed the EQ5D VAS score, where 0 = worst imaginable health and 100 = best imaginable health (EuroQol Group, 1990; King et al., 2009; Rabin, 2001).
3. The Quality of Recovery (QoR)‐15 questionnaire, which was developed by Myles et al. originally as a 40 items version (Myles et al., 2000), and down‐scaled to a 15‐items version, which also has been shown to be valid and reliable (Stark et al., 2013). The QoR‐15 consists of 15 questions distributed on two dimensions: “physical” and “mental” well‐being. The patients report their experiences on a scale from 0 (=not at all) to 10 (=all the time), where negatively loaded questions are reversed. This gives a maximum score = 150. The QoR‐15 was translated and validated in Norwegian based on preliminary findings from the current study (Leonardsen & Svensen, 2020). In the current study the Cronbach's alpha for the QoR‐15 at T1 was 0.87. At T2, Cronbach's alpha was 0.84.
4. Weight, height (calculating body‐mass‐index, BMI), ASA classification (American Association of Anaesthesiologists' classification of patients' condition), duration of surgery and duration of the whole procedure (including anaesthesia) were registered by study nurses.

### 2.6 Phase 2 questionnaire

The questionnaire in phase 2 included the QoR‐15 questionnaire and the validated questionnaire “Pasienterfaringer etter dagkirurgi” (Patients' experiences after day surgery), developed by the Norwegian Knowledge Center. This questionnaire consists of 24 questions, divided into five categories: (1) overall impression, (2) availability and reception, (3) treatment and care, (4) information and (5) experiences after discharge, as well as free‐text answers. The answers are reported on a 5‐point Likert's scale, where 1 = not at all/worse than expected, 2 = to a small extent/less than expected, 3 = to some extent/as expected, 4 = to a large degree/better than expected and 5 = very large degree/much better than expected (Holmboe et al., 2010). For the Patients' experience questionnaire, Cronbach's alpha was 0.78 in the current study.

When scoring the QoR‐15 questionnaire, patients at T2 were asked to recall how they felt the day after surgery‐ in retrospect (“How did you feel the first 24 h after surgery?”). The rationale for this was to explore whether patients rated the quality higher directly after surgery, when still receiving analgesics and when called by study nurses compared to when evaluating the quality in retrospect.

### 2.7 Analysis

Internal consistency was assessed using Cronbach's alpha. Since we used two different scales to measure patient experiences, we also conducted Spearman's rho correlation analysis between variables in the two scales.

Descriptive statistics and frequencies were used to describe the sample. Kruskal–Wallis tests (one‐way ANOVA) were used to compare participants from the three hospitals. Paired sample t‐test was used to compare QoR‐15 scores at T1 and T2. A linear regression model that used socio‐demographic variables (age, BMI, living arrangements, civil status, employment status and educational background), EQ5D index score, ASA classification, duration of surgery and procedure as independent variables and QoR‐15 score (QoR‐15 at T1) as dependent variable was used. Insignificant variables were removed from the model one at a time until only statistically significant effects remained. Missing items were not included in the analysis.

All tests were two‐sided, using a significance level below .05. All analyses were conducted using the Statistical Package for the Social Sciences (SPSS) version 26 (IBM Corporation, 2019).

## 3 RESULTS

### 3.1 Correlations between QoR‐15 and patient experiences

Spearman's rho correlation analysis showed weak, but statistically significant associations between several of the items on the QoR‐15 questionnaire and the patients' experiences questionnaire. “Waiting time” was positively correlated to all of the QoR‐15 items, ranging from .20 to .29 (*p* < .001). Whether the patients experienced to received needed information was also positively correlated to all of the items, ranging from .18 to .35 (*p* < .001). “Trust in healthcare personnel's skills” (range .16 to .35) and whether “healthcare personnel showed interest in the patients' situation” (range .14 to .29) were also positively correlated to several of the QoR‐15 items (e.g. able to breathe, enjoy food, sleep well and feel comfortable) .

### 3.2 Participants

At T1, a total of 444 participants responded to the questionnaire in the period from March 2019 to March 2020. Of these, 193 participants responded to the questionnaire at T2. Tables 2 and 3 give an overview of participants' descriptives in the three hospitals and T1 and T2 respectively.

**TABLE 2:** Descriptives of participants at T1 (*n* = 444)

	Hospital 1 (*n* = 117)	Hospital 2 (*n* = 190)	Hospital 3 (*n* = 137)	All participants (*n* = 444)	*p*‐value
Age, mean (SD)	47 (14.3)	49 (13.8)	38 (13.6)	46 (14.4)	<.01*
Age range	19–85	18–83	20–81	18–85	
Height, mean (SD)	168 (6.3)	168 (6.7)	167 (6.1)	167 (6.5)	.02*
Weight, mean (SD)	71 (19.6)	72 (16.5)	70 (17.3)	71 (17.5)	.80
BMI, mean (SD)	25.4 (6.5)	25.9 (5.4)	26 (6)	25.7 (5.9)	.60
EQ5D5L index score, mean (SD)	.75 (.25)	.70 (.23)	.74 (.22)	.73 (.23)	.60
EQ5D5L VAS, mean (SD)	80 (21.3)	80 (20.2)	80 (17.5)	80 (19.8)	.42
Surgery time, mean (SD)	25.2 (5.5)	9.3 (12.6)	8.4 (6)	10.5 (48.6)	.04*
Procedure time, mean (SD)	42 (5.5)	15.3 (12.6)	39 (24)	39 (56.4)	
ASA (%)					.55
I	40	37.2	40.1	38.8	
II	54.8	51.3	51.1	52.1	
III	4.3	11.5	8	8.6	
IV	0.9		0.7	0.5	
Civil status (%)					.82
Married	70.9	69.6	68.6	69.7	
Single	16.2	23	18.2	19.8	
Widow	6.8	3.7	0.7	3.6	
Couple	6.0	3.7	12.4	7.0	
Living alone (%)					.13
Yes	26.5	20.4	29.9	24.9	
No	73.5	79.6	70.1	75.1	
Highest educational level (%)					.61
Compulsory school	17.1	11	7.3	11.5	
Upper secondary	35.9	38.7	46	40.2	
University	47.0	50.3	46.7	48.3	
Still working (%)					.05
Yes	66.7	66	81.8	69.9	
No	9.4	11	14.6	9.7	
Retired	15.4	15.7	13.9	20.4	
Pain score, median (SD)	1 (2.5)	0 (1.9)	1 (1.9)	1 (2.1)	.06

*Note*: Age: in years. Height: in centimetres. Weight: in kilograms. BMI calculated as Weight/Height^2^. EQ5D5L VAS (Visual analogue scale): 0 = worst health, 100 = best health. EQ5D5L index score, based on the Europe VAS value set: 0 = worst health, 1 = best health. Surgery and procedure time: in minutes.

* Statistically significant differences between the three hospitals at a .05 level. Kruskal–Wallis test.

**TABLE 3:** Participants at T2 (*n* = 193)

	Hospital 1 (*n* = 37)	Hospital 2 (*n* = 93)	Hospital 3 (*n* = 63)
Age, mean (SD)	47.9 (14.2)	51.4 (13.6)	42.7 (13.3)
Age, range	20–85	20–83	21–85
BMI	26.6 (7.8)	26.6 (5.8)	27.1 (5.3)
ASA			
I	43.2	40.9	46.0
II	56.8	47.3	42.9
III	–	11.8	11.1

*Note*: Age: in years. BMI calculated as weight/height^2^.

### 3.3 Quality of recovery

Quality of recovery was scored at both T1 and T2. Table 4 gives an overview of QoR‐15 scores at T1 and T2.

**TABLE 4:** Quality of recovery (QoR)‐15 scores at T1 and T2

	Hospital 1 (*n* = 117/37)	Hospital 2 (*n* = 190/93)	Hospital 3 (*n* = 137/63)	All participants	*p*‐value
QoR‐score at T1/T2	132 (22.6)/ 124 (20.5)	132 (24.1)/ 128.5 (27.7)	134 (17.9)/ 127 (20.9)	133 (21.9)/ 127 (24.4)	.01

*Note*: QOR, item number. Total score range 0 (worst quality) to 150 (best quality). Values in mean, standard deviation in parenthesis. T1 = first postoperative day. T2 = after 4 weeks. Paired *t*‐test.

Abbreviation: QoR, quality of recovery.

The QoR‐15 score was significantly lower at T2 than at T1 (*p* = .01).

### 3.4 Patient experiences after four weeks

Table 5 gives an overview of responses to the “Patients' experiences after day‐surgery” questionnaire.

**TABLE 5:** Patient experiences at T2

	Hospital 1 (*n* = 37)	Hospital 2 (*n* = 93)	Hospital 3 (*n* = 63)	All participants (*n* = 193)
1. Satisfactory care and treatment	5 (0.8)	5 (0.5)	5 (0.5)	5 (0.6)
2. Overall evaluation	4 (0.9)	4 (0.9)	5 (0.9)	4 (0.9)
3. Waiting time for surgery	4 (1)	4 (1)	5 (0.8)	4 (0.9)
4. Easy to find the way to the unit	5 (0.8)	5 (0.9)	5 (0.8)	5 (0.9)
5. Unforeseen waiting at the day of surgery^^	2 (1.1)	1 (0.9)	2 (0.9)	1 (0.9)
6. Doctors talked so you could understand	5 (0.5)	5 (0.6)	5 (0.5)	5 (0.5)
7. Trust in doctors' professional skills	5 (0.7)	5 (0.5)	5 (0.4)	5 (0.5)
8. Nurses interested in your situation	5 (0.6)	5 (0.5)	5 (0.5)	5 (0.5)
9. Nurses cared for you	5 (0.7)	5 (0.6)	5 (0.6)	5 (0.6)
10. Doctors interested in your situation	5 (0.8)	5 (0.6)	5 (0.6)	5 (0.6)
11. Nurses interested in your situation	5 (0.7)	5 (0.6)	5 (0.6)	5 (0.6)
12. Mistreated in any way	1 (0.9)	1 (1.2)	1 (0.8)	1 (1)
13. Got enough information before admission	4 (0.8)	4 (0.8)	4 (0.9)	4 (0.7)
14. Personnel had received the information about you	5 (0.7)	4 (0.7)	4 (0.7)	4 (0.7)
15. Got the information you needed about the procedure	5 (0.8)	5 (0.8)	5 (0.8)	5 (0.8)
16. Got the information you needed about anaesthesia	5 (0.7)	5 (0.9)	5 (0.7)	5 (0.8)
17. Got information you needed about time after discharge	4 (0.9)	4 (0.9)	4 (0.6)	4 (0.9)
18. Got information you needed about effects and side‐effects of medication	5 (0.9)	4 (1.1)	4 (0.9)	4 (1)
19. Unanswered questions at discharge	1.5 (1.3)	1 (1.1)	1 (1.2)	1 (1.1)
20. Outcome of the stay in the unit‐overall	4 (1.1)	4 (1.1)	4 (0.8)	4 (1.1)
21. Experienced complications, *n* (%)				
Yes	7 (18.9)	20 (21.5)	11 (17.5)	39 (20.3)
No	30 (81.1)	73 (78.5)	52 (82.5)	154 (79.7)
22. Nature of complications, *n* (%)				
Infections	1 (14.3)	1 (7.7)	1 (9)	3 (8.5)
Haemorrhage	2 (28.6)	9 (45)	6 (54.5)	16 (42.6)
Nausea	1 (14.3)	1 (7.7)	–	3 (6.4)
Pain	3 (42.9)	9 (45)	4 (36.5)	17 (42.6)
23. Been in contact with healthcare services due to complications, *n* (%)				
Yes	3 (9.1)	4 (20)	2 (18.2)	7 (17.5)
No	20 (54.5)	5 (20)	3 (27.3)	11 (30.4)
Not relevant	14 (36.4)	11 (60)	6 (54.5)	21 (52)
24. Complications led to hospitalization, *n* (%)				
Yes	1 (3)	2 (2.4)	1 (1.8)	6 (2.9)
No	19 (60.6)	51 (54.9)	26 (41.1)	99 (51.4)
Not relevant	17 (45.9)	40 (43)	36 (57.1)	88 (45.7)

*Note*: Questionnaire from the Norwegian Knowledge Center “Patient experiences with ambulatory surgery.” Scored on a 5‐point Likert scale where 1 = not at all, 2 = small degree, 3 = some degree, 4 = large degree, 5 = very large degree (1 = most negative, while 5 = most positive), except questions^^ 5, 12 and 18, where 1 = most positive and 5 = most negative. Reported as mean scores, with standard deviation in parenthesis.

Participants' reported to be satisfied to a large or very large degree.

### 3.5 Factors influencing quality of recovery scores

“EQ5D index score” was the only independent variable that made a statistically significant contribution to the model at T1. Hence, age, BMI, living arrangements, civil status, employment status, educational background, ASA classification, duration of surgery and duration of procedure did not have any impact on patients experiences with quality of recovery. Table 6 shows the statistically significant result from the regression model analysis.

**TABLE 6:** Statistically significant result from the linear regression model

	*R* ^2^	*F*	95% confidence interval	Coefficient beta	*p*‐value
EQ5D index score	.169	82.86	.53–.99	.411	<.001

However, the effect size of this factor was limited, indicating a weak association between participants' self‐assessed health‐related quality of life (EQ5D3L) and assessment of quality of recovery as measured with the QoR‐15 at T1.

## 4 DISCUSSION

This study adds information about day surgical patients' experiences of quality of recovery and other outcome measures1 day and 4 weeks postoperatively. Our findings establish the pitfalls of day surgery, as well as highlight areas that need improvement. In addition, this study shows two different measures that both are appropriate and valid for exploring different aspects of patients' experiences after day surgery.

In our study, we found several correlations between QoR‐15 items and the tangibility aspects of quality, such as waiting times, information provision, trust in and interest from healthcare personnel. To date, postoperative quality of recovery lacks a universally accepted definition and assessment technique, and several tools to measure such quality exists (Bower et al., 2014). Hence, our findings contribute a wider picture of patient experience, including both patient‐assessed quality, patient experienced symptoms, and information about re‐admission/hospitalization and postoperative infection.

In our study, patients' experience of quality of recovery (QoR‐15) was significantly lower 4 weeks after surgery than the day after surgery. This is in line with earlier research (McGrath et al., 2004; McIntosh, 2011). During the postoperative phase, patient satisfaction is related to having pain under control, receiving appropriate postoperative information, sharing in decision‐making and being treated with respect and dignity (Jaensson et al., 2019). At T1, patients were called by study nurses, which may have supported a positive experience. It is important for healthcare personnel to know that unexpected problems in the postoperative process affect the patient's feeling of well‐being. Therefore, patients should be informed about the types of discomforting symptoms they could experience during their postoperative recovery process (Nilsson et al., 2018). When evaluating quality of recovery in retrospect, at T2, patients may have become more aware of the discomforting symptoms. Additionally, twenty per cent experienced complications such as infection, haemorrhage and pain, which may also have impacted the QoR‐15 scores.

About patients' experiences with other measures, five items proved quality improvement potential, namely “waiting time for surgery,” “unforeseen waiting at the day of surgery” (two hospitals), “got enough information before admission,” “got the information needed about the time after discharge” as well as “effects and side‐effects of medication.” The provision of sufficient pre‐operative information pre‐operatively can increase patient satisfaction (Mitchell, 2013, 2015). The mode and timing of the provision of pre‐operative information require careful consideration of patient participation, and the information strategies used should be optimal. Some patients even report experiencing heightened stress and anxiety while visiting the hospital for pre‐operative information and assessment (Alanazi, 2014). Still, a lack of sufficient information can cause uncertainty and anxiety for the patient (Berg et al., 2013). It is challenging for a healthcare provider to meet patients' expectations about the amount and type of information needed (Sibbern et al., 2017).

As much as 20 per cent of the participants reported to have experienced complications after surgery, such as infection (8.5 per cent), haemorrhage (42.6 per cent), nausea (6.4 per cent) and pain (42.6 per cent). Of these, 17.5 per cent had been in contact with healthcare services, leading to hospitalization in 2.9 per cent. We do not know the reason why patients did not contact healthcare personnel, whether this was due to weak symptoms or if it was due to the lack of knowledge about what is normal after surgery. It would be appropriate to assume that patients experiencing complications had less positive experiences. In a recent Norwegian study, the only negative association with patient experiences (with having a piccline or a midline/venous access for long‐term intravenous treatment) was the occurrence of complications (Leonardsen et al., 2020). Still, earlier studies have provided contradictory results on the association between complications and patient experiences. For example, Sacks et al. (2015) and Prabhu et al. (2018) found statistically significant associations between patient satisfaction and the occurrence of postsurgical complications. Others claim that factors such as high surgical volume, rather than postoperative complications, are associated with patient dissatisfaction (Kennedy et al., 2014; Tevis & Kennedy, 2015). Our findings stress the importance of promoting realistic expectations of the recovery process not only to manage inconvenience or discomfort, but also to be aware of normal recovery problems and complications. Hence, to support an adequate mental and practical preparation for the postoperative recovery, an optimal information structure and dissemination needs to be identified.

The only association we identified was between EQ5D index score and the experience of quality of recovery at T1, and the effect of this was weak. This is in contrast to other studies that found associations between patient satisfaction and, for example “type of surgery” (Stessel et al., 2015), “young age” and “employment status = not working” (Brix et al., 2017), and “duration of the procedure,” “female gender” and “laparoscopic cholecystectomy” (Stessel et al., 2015). Our study included women undergoing gynaecological procedures only. Koenig (2018) has reported that people believe men tend to show less emotion than women, and that gender role stereotypes may be less relevant in older age groups. It is possible that including both gender, and/or another type of surgery may have provided different results. This needs to be further explored.

The aim of healthcare services is to provide a high quality of care. One way to ensure that this aim has been fulfilled is to assess not only patients' satisfaction, but also patients' experiences with their care. There are different ways how to interpret satisfaction in patients (Berkowitz, 2016). In a concept analysis of patient satisfaction, four attributes have been identified: the provider's attitude, the provider's technical competence, the accessibility of health care and the efficacy of health care (Ng & Luk, 2019). Based on our findings, this is transferable to patients' experiences.

Undergoing day surgery puts demands on patients to self‐manage their postoperative recovery at home (Berg et al., 2013). The importance of the family caregiver role during the perioperative period, including recovery at home, has been reported in the literature, and the responsibility of family members at home has increased with the rise in day surgery (Dawe et al., 2014). This underlines the importance on quality improvement initiatives, to ensure an optimal day surgical pathway for patients.

### 4.1 Strengths and limitations

Sample size calculations indicated a need for 100 patients from each hospital. We did not reach this number of participants during the data collection period. In addition, only approximately half of the participants responded to the T2 questionnaire. This may limit the reliability and generalizability of our findings. Nevertheless, data were collected from three different hospitals, including patients with a range in age and other demographics, gynaecological procedures and length of surgery, which may increase the transferability of our findings. The questionnaires showed good internal validity and also showed to complement each other.

In all hospitals, dedicated study nurses were responsible for interviewing the patients 24 h postoperatively. This may also increase the validity of our findings at T1. However, at T2 patients completed the questionnaires themselves. Still, we found associations between responses to the QoR‐15 and the patient experience questionnaire, which support the validity of our findings also at T2.

Due to the nature of the chosen surgical procedures, we only included females in this study. This implicates that our findings may not be transferred to a male population, or to other surgical procedures. Hence, our findings support the need to conduct further research on quality of recovery, patients' experiences and complications after day surgery longitudinally.

## 5 CONCLUSION

Participants reported lower quality of recovery in retrospect 4 weeks after surgery than the first day postoperative. This underlines the importance of taking conscious choices about when patient experience is collected in studies. Participants reported few negative experiences after day surgery. Nevertheless, this study identified areas that should be focused on in quality improvement initiatives. When planning future healthcare services, with an increased focus on day surgery rather than hospitalization, these aspects are important to include in the process. More studies on patients' experiences with day surgery, rather than satisfaction, are needed. Our study indicates that it is appropriate to combine different measures when exploring patient experiences after day surgery. Hence, our study should be repeated in other surgical procedures including both genders.

## FUNDING INFORMATION

The study was funded by the Norwegian Nurses' Association and collaborative fundings from Østfold University College. None of the funders participated in planning or conducting the study, or analysing and disseminating the findings.

## CONFLICT OF INTEREST

AMGB, KS, EJ, TDH, AG and ACL declared no competing interests.

## ETHICAL APPROVAL

Consent was granted from the Norwegian Committee for Medical and Health Services Research (REK, project no. 2018/985), and the Norwegian Centre for Research Data (NSD, project no. 416326).

## Data Availability

Data is available upon reasonable request to the corresponding author
